# Correlation of PET-CT, MRI and histopathology findings in the follow-up of patients with nasopharyngeal cancer

**DOI:** 10.1016/j.bjorl.2019.12.004

**Published:** 2020-01-13

**Authors:** Semih Ak, Caner Kiliç, Samet Özlügedik

**Affiliations:** a Şanlıurfa Mehmet Akif Inan Training and Research Hospital, Department of Otorhinolaryngology, Sanlıurfa, Turkey; b University of Health Sciences Ankara Dr. Abdurrahman Yurtaslan Oncology, Department of Otorhinolaryngology, Ankara,Turkey

**Keywords:** Nasopharynx cancer, PET-CT, MR, Histopathology

## Abstract

**Introduction:**

Surgical treatment options are limited for nasopharyngeal cancer for many reasons including epidemiological and histological properties, proximity to important structures, heavy lymphatic drainage, and the difficulty in ensuring a safe surgical margin; therefore primary treatment is generally radiotherapy and chemotherapy. With current radiotherapy technology, oncological success has been increased and the quality of life of patients during the post- radiotherapy period is improved.

**Objective:**

The role of magnetic resonance imaging and positron emission-computed tomography in the follow-up of recurrent nasopharyngeal cancer patients who were initially treated with radiotherapy was evaluated with respect to histopathological findings.

**Methods:**

A total of 110 patients with nasopharyngeal cancer who had received radiotherapy were included in the study. Patients who were suspected to have recurrence according to endoscopic nasopharyngeal examination and magnetic resonance imaging findings were requested to undergo positron emission-computed tomography. Biopsies were taken from 40 patients who had suspicious lesions in positron emission-computed tomography images. These patients’ age, gender, presence/absence of contrast enhancement on magnetic resonance imaging, the SuvMax values of nasopharyngeal and neck lesions, T/N phases at initial diagnosis, histopathological recurrence, and history of neck dissection were assessed.

**Results:**

Recurrence was observed in 8 patients (20.0%). Among these, 4 (10.0%) had recurrence at the nasopharynx and 4 (10.0%) at the neck. Patients with recurrence were found to be of older age, male gender, advanced T/N phase, contrast enhancement on magnetic resonance imaging, and higher nasopharyngeal and neck SuvMax values in positron emission-computed tomography. However, these differences were not statistically significant. Only the history of neck dissection was significantly more common among those with recurrence (*p* < 0.001). However, in multivariate analysis, those with a nasopharyngeal SuvMax value higher than 4.58 were found to have 7.667-fold higher risk for recurrence (*p* = 0.036).

**Conclusions:**

Magnetic resonance imaging and positron emission-computed tomography should be evaluated together in the follow-up of nasopharyngeal cancer. Patients with minimal SuvMax 4.58 on positron emission-computed tomography after contrast enhancement in the T2 sequence on magnetic resonance imaging may considered appropriate for biopsy. Biopsies in patients with a SuvMax value lower than 4.58 can be avoided. Thus, patients avoid surgical stress and unnecessary costs.

## Introduction

Surgical treatment options are limited for nasopharyngeal cancer (NPC) for many reasons including epidemiological and histological properties, proximity to important structures, heavy lymphatic drainage, and the difficulty in ensuring a safe surgical margin; therefore primary treatment is generally radiotherapy (RT) and chemotherapy (CT). With current RT technology, oncological success has been increased and the quality of life of patients during the post-RT period is improved. The developments in computed tomography and magnetic resonance imaging (MRI) techniques have resulted in a more accurate determination of the tumor's 3D/4D structure, enabling better calculation of the dosing and focusing of RT.^1^ These developments in radiotherapy have increased success in recurrent as well as primary cases. Therefore, accuracy in the recognition of recurrence is of high importance in terms of treatment success and survival outcomes.^2^, ^3^

Mucosal changes caused by radiotherapy may lead to difficulties in the diagnosis of recurrence. Computed tomography and 18-fluoro-2-deoxi-glucose (18f-FDG) positron emission tomography (PET-CT) both play an important role in the evaluation of primary and recurrent NPC after its staging and treatment. MRI is an invaluable diagnostic tool due to its ability to evaluate soft tissue with ideal contrast and resolution without reliance on radioactivity. However, it is not always reliable in the evaluation of residual disease, recurrence and post-chemoradiotherapy fibrosis.^4^ 18f-FDG PET/CT imaging is a technology that eliminates the need for plain radiography, ultrasound, (CT) and bone-scan combinations. This mode of examination also provides anatomical and functional information. Due to its high positive predictive values in the detection of recurrence and metastasis, it is known to contribute to the planning of treatment, monitoring of recurrence and survival.^5^

In this study, we evaluated the role of MRI and PET-CT in NPC cases treated with RT and compared the findings with histopathological results.

## Methods

The PET-CT, MRI and histopathological data of patients who received RT for NPC was retrospectively evaluated. Permission was obtained for this study in the ethical meeting decision dated 24.11.2017 and numbered 23. A total of 110 patients with NPC diagnosis were included in the study, and all patients underwent a control nasopharynx MRI. PET-CT was requested from patients who had suspicious lesions in endoscopic nasopharyngeal examination and those with increased intensity in MRI. Biopsy was obtained in 40 patients with suspicious lesions on PET-CT.

In endoscopic examination; nasopharynx mucosal vegetan (*meaning here is unclear*) formation or submucosal asymmetry and fullness was considered as suspicious lesion. The presence of contrast enhancement in T2 in nasopharynx and neck lymphadenopathy in MRI and Also the limitation of diffusion were considered as a suspicious lesion.

In our study group, 15 patients (37.5%) were female while 25 (62.5%) were male. One (2.5%) of the cases was at T1, 19 (47.5%) at T2, 16 (40.0%) at T3 and 4 (10.0%) were at the T4 phase. In addition, 5 patients (12.5%) had N0, 16 (40.0%) had N1, 18 (45.0%) were N2, while 1 (2.5%) were at the N3 neck stage.

Patients had received RT with one of the following techniques: simultaneous Integrated Boost technique (density adjustable radiotherapy), Vmat (volumetric arc therapy) or HT (helical tomotherapy) therapy. Local advanced stage disease (T1 N1-3, T2-T4 any N) patients underwent a definitive chemoradiotherapy treatment with additional cisplatin 40 mg/m^2^ chemotherapy. After concomitant therapy was ended, patients were given 3 cures of consolidation treatment in the form of cisplatin/5-Fluorouracil combination chemotherapy.

The age, gender, MRI, presence of contrast-holding lesion, the SuvMax values of lesions in the nasopharynx and neck according to PET-CT, T phase at initial diagnosis, N stages (Primary stage), histopathological recurrence, neck dissection status of all 40 patients included in the study were evaluated statistically. Patients with NPC were evaluated for MRI and PET-CT findings and the associations between these findings and histopathological results were determined. The determination of the PET-CT SuvMax measurements was performed by calculating the area below the ROC curve (Area Under Curve ‒ AUC) and 95% Confidence Intervals.

PET-CT was performed in patients with contrast agent involvement and/ or necrosis in the lymph node on MRI. Patients with high SuvMax were diagnosed as malignant by USG guided biopsy.

All patients were followed up every 3 months for the first 2 years and every 6 months until the 5th year.

### Statistical analysis

Analysis of data was performed with the IBM SPSS Statistics 17.0 (IBM Corporation, Armonk, NY, USA) package program. *P*-values ≤ 0.05 were considered statistically significant. The best cut-off points for PET-CT SuvMax measurements were determined by using the Youden index (the point at which the total of sensitivity and selectivity values reached the maximum), given that the remaining area under the curve was statistically significant. The diagnostic performances of the PET-CT and MRI results were evaluated by calculating sensitivity, selectivity, positive and negative predictive values (PPV and NPV), and diagnostic accuracy rates. All variables identified to have a *p*-value < 0.25 according to univariate analyses were included in the multivariate logistic regression model for the determination of risk factors. In addition, the odds ratio for each variable, the 95% confidence interval and the Wald statistics were calculated.

## Results

Data of 40 cases with ages ranging from 26 to 74 years were evaluated in the study. The mean age of the patients was 48.5 ± 12.4 years. In 8 cases (20.0%), pathological findings showed development of recurrence. Among these, 4 (10.0%) were located in the nasopharynx while, 4 (10.0%) were in the neck. In the recurrence group; older age, male gender, advanced T and N stage, presence of contrast enhancement in MRI, and increased SuvMax levels in the nasopharynx and neck were observed; however these differences were not statistically significant. Only having undergone a neck dissection was found to be a statistically significant risk factor (*p* < 0.001) (Table 1). In patients with recurrence, the area below the ROC curve for PET-CT SuvMax-nasopharynx measurements was statistically significant (AUC = 0.719 and 95% Confidence Interval: 0.509‒0.929). The best cut-off point for PET-CT SuvMax-nasopharynx measurements was found to be 4.58 for the detection of recurrence (*p* = 0.036) (Table 2). PET-CT imaging was found to have a sensitivity of 75.0%, specificity of 71.9%, PPV of 40.0%, NPV of 92.0% for recurrence detection, while diagnostic accuracy was 72.5%.

**Table 1:** Demographic and clinical features of patients according to groups.

	No recurrence (n = 32)	Recurrence (n = 8)	*p*-Value
Age (years)	46.4 ± 12.1	56.7 ± 10.6	0.033^a^
Gender			0.686^b^
Female	13 (40.6%)	2 (25.0%)	
Male	19 (59.4%)	6 (75.0%)	
Neck dissection			< 0.001^b^
No	32 (100.0%)	4 (%50.0)	
Yes	0 (0.05%)	4 (%50,0)	
N staging			0.475^c^
0	5 (15.6%)	0 (0.0%)	
1	12 (37.5%)	4 (50.0%)	
2	15 (46.9%)	3 (37.5%)	
3	0 (0.0%)	1 (12.5%)	
T staging			0.197^c^
1	1 (3.1%)	0 (0.0%)	
2	17 (53.1%)	2 (25.0%)	
3	11 (34.4%)	5 (62.5%)	
4	3 (9.4%)	1 (12.5%)	
Contrast enhancement on MRI			0.563^b^
No	5 (15.9%)	0 (0.0%)	
Yes	27 (84.4%)	8 (100.0%)	
PET-CT SuvMax NFX	3.2 ± 2.7	5.0 ± 2.6	0.060^c^
PET-CT SuvMax neck	1.2 ± 1.9	1.4 ± 2.2	0.908^c^

a Data has been expressed as mean ± standard variation, Student's *t*-test.

b Data has been expressed as number of cases and percentages, Fisher's definite probability result test.

c Data has been expressed as mean ± standard variation, Mann–Whitney U test.

**Table 2:** Diagnostic performance Indicators of PET-CT SuvMax and MRI findings in the detection of recurrence.

	PET-CT NFX	PET-CT neck	MRI
Area under the curve	0.719	0.514	‒
95% Confidence Interval	0.509‒0.929	0.281‒0.746	‒
Best cut-off point	>4.58	‒	‒
Sensitivity, [TP/(TP + FN)]	6/8 (75.0%)	‒	8/8 (100.0%)
Specificity, [TN/(TN + FP)]	23/32 (71.9%)	‒	5/32 (15.6%)
PPV, [TP/(TP + FP)]	6/15 (40.0%)	‒	8/35 (22.9%)
NPV, [TN/(FN + TN)]	23/25 (92.0%)	‒	5/5 (100.0%)
Accuracy, [(TP + TN)/(N)]	29/40 (72.5%)	‒	13/40 (32.5%)
*p*-value^a^	0.036	‒	0.563

TP, True Positive; FN, False Negative; TN, True Negative; FP, False Positive; N, Total number of cases; PPV, Positive Predictive Value; NPV, Negative Predictive Value.

a Fisher's definite result probability test.

There were no statistically significant correlations between PET-CT SuvMax measurements and the presence/absence of lesions with contrast uptake on MRI (Table 3).

**Table 3:** PET-CT findings of the patients according to MRI enhancement in groups with and without lesions.

	Lesion not present (n = 5)	Lesion present (n = 35)	*p*-Values
PET-CT SuvMax NFX	1.3 ± 2.4	3.9 ± 2.7	0.078^a^
PET-CT SuvMax NFX			0.633^b^
<4.58	4 (80.0%)	21 (60.0%)	
>4.58	1 (20.0%)	14 (40.0%)	
PET-BT SuvMax neck	1.0 ± 1.7	1.3 ± 2.0	1.000^a^

a Data has been expressed as mean ± standard variation, Mann–Whitney U test.

b Data has been expressed as number of cases, Fisher's definite result probability test.

In multivariate analysis, the “T stage” was found to have the least importance in distinguishing recurrence among cases and was omitted after the first step. In the next step, the age variable was found to be the least effective and was also omitted. In the final model only the PET-CT SuvMax- nasopharynx variable was used, and comparisons according to the 4.58 cut-off revealed that those with a PET-CT SuvMax value higher than 4.58, had 7.667 fold higher risk for recurrence compared to those with PET-CT SuvMax values lower than 4.58 (95% CI: 1.298‒45.289; *p*  = 0.025) (Table 4).

**Table 4:** Detection of the most detecting the most decisive factor (s) in differentiating the recurrence and non recurrence groups with a multi-variate retrospective, logistic regression analysis.

	Odds ratio	95% Confidence Interval		Wald	*p*-Values
		Lower limit	Upper limit		
Model 1					
Age	1.087	0.974	1.212	2.213	0.137
T staging	2.452	0.576	10.443	1.471	0.225
PET-CT SuvMax Nfx > 4.58	4.006	0.570	28.151	1.946	0.163
Model 2					
Age	1.066	0.977	1.164	2.067	0.150
PET-CT SuvMax Nfx > 4.58	5.186	0.801	33.592	2.982	0.084
Model 3					
PET-CT SuvMax Nfx > 4.58	7,667	1,298	45,289	5,052	0.025

## Discussion

With the increase of radiotherapy and chemotherapy possibilities in patients with nasopharyngeal cancer, 5 year survival rates have increased and currently stand at 80%.^7^ However, 15%–58% of patients develop relapse and require further treatment.^6^, ^7^ Although a consensus has been reached for the treatment of primary disease, options are limited in those with recurrence. Re-irradiation is currently seen as the most viable treatment course in such patients; however, it must be carried out with careful consideration of toxicity.^8^

Male/female ratio of recurrence development has been reported as 4:1, while the same ratio is 2:1 for primary tumors.^9^ In our study, there was no difference in the distribution of recurrence between men and women according to pathology results (*p* = 0.686). The duration between initial treatment and recurrence has been shown to vary significantly; Lee et al.^10^ have identified the median latent period as 1.9 years, and they also reported that shorter latency was associated with shorter survival. In the light of this information, we can conclude that follow-up studies during the post-RT period should be performed at short intervals in order to identify recurrence at an early stage. However, there currently is no consensus on the type and technique of imaging methods which must be used during follow-up.

Although the gold standard diagnostic method is biopsy, the most commonly used method is MRI, owing to its ease of application. In patients with mucosal recurrence, direct visual inspection of the area via flexible endoscopy is the most sensitive method. However, the detection of submucosal or deep-seated tumors is very difficult. Furthermore, mucositis and scars that occur after radiotherapy cause difficulties in the visualization of the area.^11^ It has been reported that flexible endoscopy was inconclusive and diagnosis was made with MRI images in around 28% of recurrent cases.^12^, ^13^

Although it is evident that MRI is greatly beneficial in the diagnosis of primary disease, it has also been reported to be very useful in showing nodal metastases.^14^, ^15^ Xu et al.^16^ used diffusion MRI imaging methods in the monitoring of 83 nasopharyngeal cancer patients treated with radiotherapy. They reported that MRI could provide quantitative and qualitative information at the cellular level, and also may play an important role in the differentiation of local fibrosis and RT damage. In our study, we also found that the incidence of contrast enhancement in MRI was higher among patients who were shown to have recurrence; the difference, however, was not statistically significant (*p* = 0.563).

Positron emission tomography is a method that provides anatomical and functional data, which eliminates the need for a combination of imaging methods such as plain radiography, ultrasonography, computed tomography and bone screening.^17^ Chen et al.^18^ in a study comprised of 70 NPC patients, compared PET-CT and CT with TNM staging. The PET-CT method was found to be significantly superior in terms of sensitivity, specificity, PPV and NPV. Additionally, PET-CT has also been found to be superior to chest radiography, abdominal ultrasound and skeletal scintigraphy in the staging of distant metastases, with high sensitivity (70%–80%) and PPV (>90%).^19^ A meta-analysis study by Liu et al.^20^ reported that PET-CT's sensitivity (95%) and specificity (90%) were higher than MRI (78%‒76%). Similarly, there are studies that indicate that PET-CT is superior to MRI in the evaluation of early treatment response.^21^, ^22^ However, a study by Comoretto et al.^14^ reported that MRI was more accurate than PET-CT in the determination of recurrence among patients with NPC (92.1% vs. 85.7%).

In our study, the average PET-CT SuvMax level was detected to be higher in those with recurrence (*p* = 0.060), and AUC of the ROC curve for PET-CT SuvMax measurements was found to be significant in differentiating recurrence among NPC patients. The best cut-off point for PET-CT SuvMax measurements was determined as 4.58, for regional recurrence at the nasopharynx. PET-CT, allows the measurement of metabolic parameters which aid clinicians in the interpretation of the tumor's biological behavior and determination of possible outcomes, metabolic tumor volume and Metabolic Index (metabolic tumor Volume × SuvMax) have been reported to have a direct correlation with survival and clinical outcome. According to these results, when the metabolic index is divided with the tumor volume, the SuvMax cut-off value is obtained. Various studies have reported different SuvMax cut-off values: Xie et al. estimated SuvMax cut-off as 4.333.^23^ while Chan et al. determined the cut-off as 7.5.^23^ In our study, the detection of recurrence in regard to T-staging did not result in statistically significant findings. However, having a history of neck dissection was associated with higher risk for recurrence.

In multivariate analyses, T staging and age were excluded due to low discrimination, leaving the PET-CT SuvMax-nasopharynx variable as the sole risk factor for recurrence. Accordingly, those with a PET-CT SuvMax value higher than 4.58, were found to have a 7.667 fold higher risk for recurrence compared to those with lower values (*p* = 0.025).

## Conclusions

The results of our study indicate that PET-CT is more effective than MRI in the determination of relapse in the primary tumor region in patients with NPC. We recommend that MRI imaging should be performed at short intervals after NPC treatment for standard follow-up studies, while PET-CT should be utilized in those with clinical suspicion for recurrence. In patients with SuvMax values higher than 4.58, biopsy should be performed. Thus, we can reduce unnecessary invasive procedures and stress caused by these procedures.

## Ethical approval

All procedures performed in studies involving human participants were in accordance with the ethical standards of the institutional and/or national research committee and with the 1964 Helsinki declaration and its later amendments or comparable ethical standards.

## Conflicts of interest

The authors declare no conflicts of interest.
